# Detection of dengue using PAMAM dendrimer integrated tapered optical fiber sensor

**DOI:** 10.1038/s41598-019-49891-7

**Published:** 2019-09-17

**Authors:** Yasmin Mustapha Kamil, Sura H. Al-Rekabi, Mohd Hanif Yaacob, Amir Syahir, Hui Yee Chee, Mohd Adzir Mahdi, Muhammad Hafiz Abu Bakar

**Affiliations:** 1 Wireless and Photonic Networks Research Centre, Faculty of Engineering, Universiti Putra Malaysia, 43400 Serdang, Malaysia; 2 Electrical Engineering Department, Al Suwayrah Technical Institute, Middle Technical University in Baghdad, Baghdad, Iraq; 3 Department of Biochemistry, Faculty of Biotechnology and Biomolecular Sciences, Universiti Putra Malaysia, 43400 Serdang, Malaysia; 4 Department of Microbiology and Parasitology, Faculty of Medicine and Health Sciences, Universiti Putra Malaysia, Serdang, 43400 Serdang, Malaysia

**Keywords:** Biomedical engineering, Optical sensors

## Abstract

The exponential escalation of dengue cases has indeed become a global health crisis. This work elaborates on the development of a biofunctionalized tapered optical fiber (TOF) based sensor with the integration of polyamidoamine (PAMAM) dendrimer for the detection of dengue E protein. The dimension of the TOF generated an evanescent field that was sensitive to any changes in the external medium while the integration of PAMAM promoted more adhesion of bio-recognition molecules; anti-DENV II E protein antibodies; that were complementary to the targeted protein. This in return created more active sites for the absorption of DENV II E proteins onto the tapered region. The resolution and detection limit of the sensor are 19.53 nm/nM and 1 pM, respectively with K_d_ = 1.02 × 10^−10^ M.

## Introduction

The escalating incidence of dengue in most parts of the globe has become more daunting in the recent decade. The arthropod-borne disease was estimated by the World Health Organization to hit 390 million victims and 25 000 fatalities annually, while putting half of the world’s population at risk of infection^1^. The culprit behind the rapid spread of the infection is the *Aedes egypti* mosquito which is capable of transmitting all four dengue strains (DENV I, DENV II, DENV III, and DENV IV). Once the virus enters a victim’s body, the individual will present high fever and flu-like illness, accompanied with severe headache, muscle and joint pains, nausea and swollen glands^2^. For severe dengue cases, symptoms may progress to plasma leakage, respiratory distress, extensive bleeding and organ dysfunction within the first 3–4 days of infection which lead to deadly complications like dengue shock syndrome and dengue hemorrhagic fever. Despite the severity of the disease, a cure for the infection is still absent, leaving victims with no option but to rely greatly on the conventional dengue diagnostics.

As the infection results in a broad spectrum of symptoms, diagnosis based on clinical symptoms alone are deemed unreliable and not specific. Thus, early laboratory confirmation during day 0–4 of the infection is very essential and may be life-saving^3–5^. Today, conventional lab-based dengue diagnostic techniques are designed to be virologically and serologically oriented. This is because viral and immunological parameters closely define the progress of infection. For example, the best determinant to detect after the onset of illness is the virus itself as it will be present in the patient’s blood and selected tissues for approximately 3 days. Among the common techniques used for detection during this crucial phase are real time polymerase chain reaction (RT-PCR) and viral isolation for the detection of viral genes and structural components^6^. However, despite the significant accuracy and sensitivity of the two techniques, both are complex to perform, very expensive and time-consuming. Also, they require specialized facilities to conduct the tests which may only be available in centralized hospitals and private diagnostic laboratories. This would incur extra cost and time wastage which create unnecessary obstructions and delay. Sadly, such delays have been reported to be the major contributor to deaths caused by the virus. Hence, there is a dire need of an improved cost-effective dengue diagnostic method that could deliver reliable sensing performance within a shorter amount of time.

Tapered optical fiber (TOF) based sensors are riding today’s trend-wave in sensor designs, promoting seamless sensing system solutions with exceptional sensitivity and selectivity. While the uniform cylindrical structure of an optical fiber is intended to propagate light with minimal loss, the tapering process allows the exposure and interaction of evanescent waves with the external medium which create the basic sensing mechanism of TOF sensors. Interferometric effect within the taper yields consistent power-independent wavelength-based detection output. The sensor is also simple to fabricate and can be operated without complex setup^7–10^. In recent research studies, TOF has been implemented in various bio-sensing systems and showed good sensing performance^9,11–15^. For dengue specifically, we have reported a bio-functionalized TOF sensor for the detection of the E proteins on DENV which achieved lower detection limit compared to other reported studies and conventional dengue diagnostics. Albeit the encouraging results, the sensing performance of TOF sensor can be further improved with the integration of nanomaterials. In the biomedical field, nanomaterial known as polyamidoamine (PAMAM) dendrimer has found themselves to be very useful for *in-vivo* and diagnostic related applications^16^. These hyper-branched macromolecules have the ability to harbour biomolecules at their periphery through polar functionalities due to the abundance of carboxyl and amino functional groups at the terminal end of the branches. Due to this feature, implementing and integrating PAMAM dendrimer into the bio-functionalized TOF as an active layer may increase the active sites of the sensor where antibody molecules can bind onto during immobilization. One study demonstrated the use of dendrimer matrices to enhance the sensing performance of an absorption-based fiber optic sensor^17^. Instead of tapering the fiber to amplify the evanescent field, fibers were bent in a U-shape instead to obtain a diameter of 1.5 mm. The study concluded that the loading efficiency of bio-molecules onto PAMAM is almost two-fold when compared to conventional surface. Intensity-based sensors like the U-shaped fiber, however, are power level-dependent thus require complementary self-referencing method to ensure its accuracy^18^. Additionally, as the intensity of evanescent wave is influenced by the bending diameter, complex setup was often needed to maintain mechanical stability^17^.

In this work, a PAMAM-integrated TOF sensor is reported. The selectivity of the sensor is ensured by immobilizing anti-DENV II E protein antibodies as bio-recognition molecules. Here, PAMAM is utilised to increase the absorption of antibodies to provide more active sites for the attachment of DENV II E proteins. It is hypothesized that this trait would increase the sensor’s sensitivity towards the antigenic determinant and consequently contribute greatly to the advancement of dengue diagnostics.

## Results and Discussion

### Characterization of PAMAM on TOF

**Figure 1:**
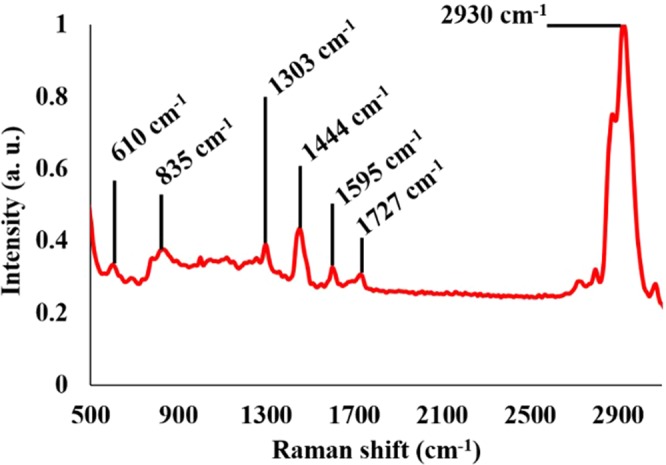
Raman spectrum after deposition of PAMAM.

The presence of PAMAM was confirmed through Raman spectroscopy (Fig. 1). Vibrational modes representing C-C and C-H bonds were apparent in the spectrum. This included band peaks 610 cm^−1^, 835 cm^−1^, and 2930 cm^−1^ ^19–21^. Aside from that, amino functional group terminals were also prominent here which were assigned to band peaks 1444 cm^−1^, 1595 cm^−1^, and 1303 cm^−1^ ^22,23^. The observation was in accordance to the attachment of PAMAM as PAMAM molecules have many amino functional groups terminating their molecular branches. On the other hand, a slight band peak at 1727 cm^−1^ was observed which may represent the remaining carboxylic ends of the glutaraldehyde on the TOF^24^.

**Figure 2:**
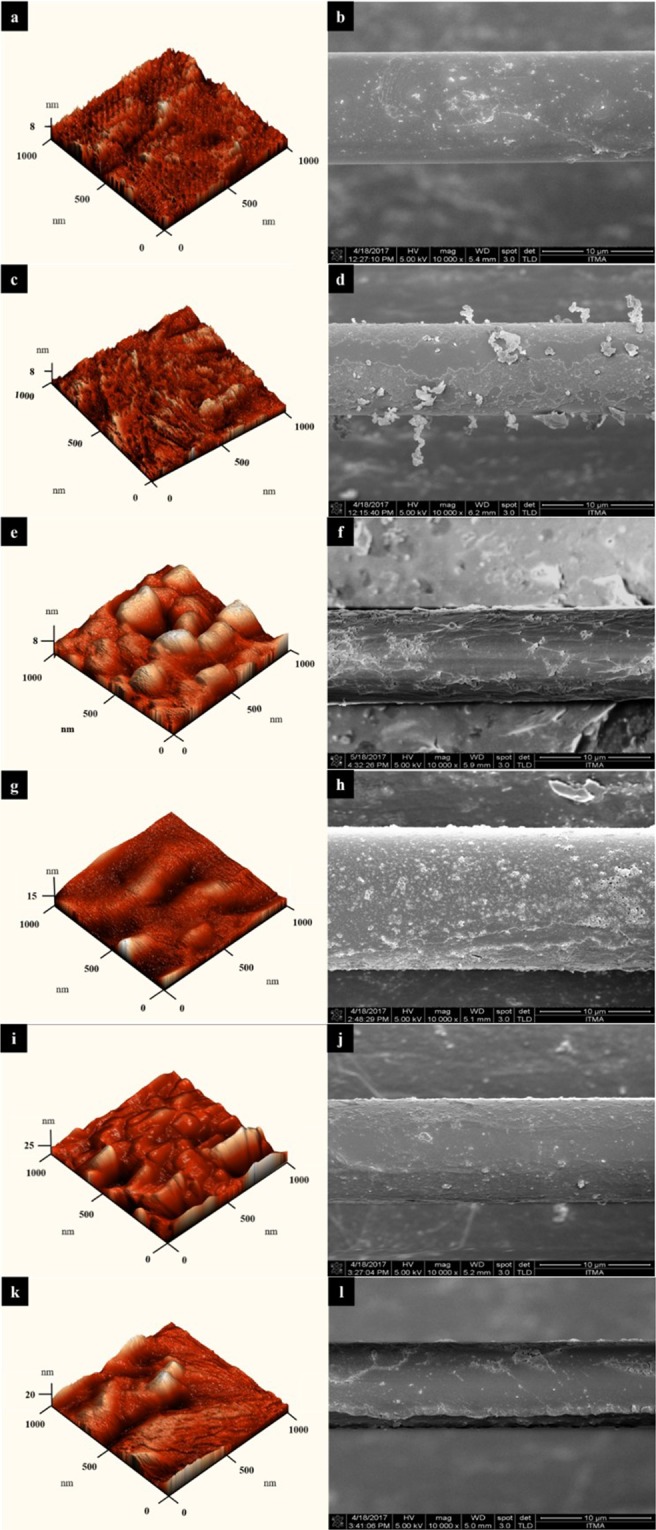
AFM (left) and FESEM (right) images of PAMAM TOF: P_1_ = (**a**,**b**); P_2_ = (**c**,**d**); P_3_ = (**e**,**f**); P_4_ = (**g**,**h**); P_5_ = (**i**) and (**j**); P_6_ = (**k**,**l**).

Characterization was continued with atomic force microscope (AFM) and field emission scanning electron microscopy (FESEM) analysis to determine the thickness, roughness, and conformation of the layers on each TOF profile tested Fig. 2(a) – (l). In this study, we have studied 6 different taper profiles; P_1_, P_2,_ P_3_, P_4_, P_5_ and P_6_; of which each profile was deposited with PAMAM at different incubation time. Here, an expected observation of thickness increment was noted as the immersion time was increased. P_1_ with an immersion time of 10 mins yielded the lowest thickness value of 8.65 nm, followed by P_2,_ P_3_, P_4_, P_5_ and P_6_ with 12.36 nm, 16.40 nm, 21.10 nm, 23.60 nm and 48.42 nm, respectively, with immersion times 20 mins, 30 mins, 40 mins, 50 mins and 60 mins. Root mean square (RMS) values which portray the roughness of the surface also showed a linear relationship with the immersion time where P_1_ yielded the lowest RMS value at 1.10, while P_6_ resulted in 6.47. In the FESEM images, as immersion time was increased, a homogenous coverage of the PAMAM molecules onto the TOF was observed. However, deformation was noted for P_5_ and P_6_. This may be caused by excessive polymerization of PAMAM on the tapered surface due to long immersion time.

### Characterization of PAMAM-integrated bio-functionalized TOF and detection of DENV II E proteins

**Figure 3:**
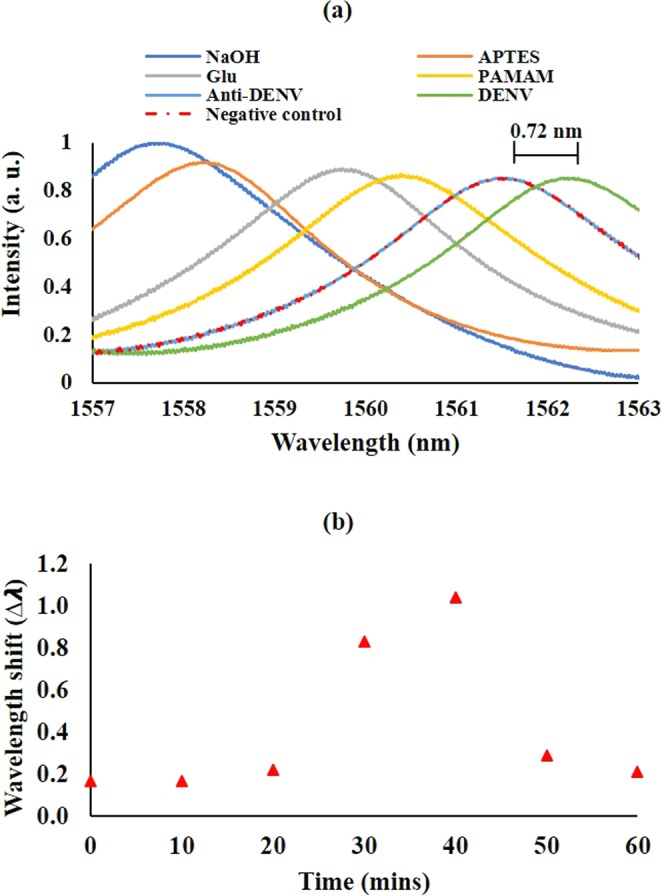
(**a**) Spectra taken after each surface functionalization step on PAMAM TOF, detection of DENV II E proteins (green line) and also with a negative control (red-dotted line) and (**b**) wavelength shift obtained for control and each taper profile when anti-DENV II E protein is introduced.

Figure 3(a) shows a consistent red shift after each surface functionalization step supporting the addition of layers. A 0.76 nm red shift was observed when the TOF was first introduced to APTES after hydroxylation. Following that, the spectrum shifted another 0.98 nm to the right after the activation process with glutaraldehyde as a response to the attachment of glutaraldehyde molecules on the TOF surface. Next, the spectrum was pushed further to the right when PAMAM was added and also after the immobilization of anti-DENV II E protein antibodies with a shift of 0.94 nm and 1.04 nm, respectively. The observations stated was mainly due to the increment of cladding refractive index which brought upon the red shift similar to the behavior reported in previous studies^8,25^. This phenomenon can be explained by looking at the transmission equation of a tapered fiber which is defined as^26^:

$$I={I}_{1}+{I}_{2}+2\sqrt{{I}_{1}{I}_{2}}cos(\Delta \Phi )$$

where *I*_1_ and *I*_2_ are light intensities of two modes which couple together as a result of the tapered dimension to produce *I*. $\Delta \Phi$ is the phase difference between the two modes which can be further elaborated as^26^:

$$\Delta \Phi =[\frac{2\pi (\Delta {n}_{eff})L}{\lambda }]$$

where $\Delta {n}_{eff}$ is the effective refractive index difference between the core and cladding of the fiber along the tapered waist length, L, at input wavelength, $\lambda$. Hence, an increment of cladding refractive index will affect $\Delta {n}_{eff}$ and subsequently $\Delta \Phi$, resulting in the red shift observation.

Figure 3(b) on the other hand shows the wavelength shift when the same concentration of anti-DENV II E protein was introduced to control (TOF without PAMAM) and all taper profiles that were tested in this work. From the figure, all PAMAM TOFs produce higher wavelength shift compared to control which emphasizes the enhancement of antibody load onto the sensor with the presence of PAMAM. This is expected as the abundance of carboxyl and amino functional group at the terminal branches of the PAMAM molecule create more sites for antibodies to react and bind, which directly increases the antibody load^17^. Among the profiles, P_4_ attained the highest wavelength shift at 1.04 nm. A sharp decline was observed afterwards as the thicker PAMAM layer limited the evanescent wave interaction with the bound antibodies.

The presence of DENV II E proteins yielded a distinguishable red shift of 0.72 nm Fig. 3(a). This is relevant to the assumption that there has been an addition of DENV II E protein molecules onto the TOF, a result from the specific affinity anti-DENV II E protein antibodies have towards DENV II E proteins, which resulted in an immunological interaction and the attachment of the DENV II E proteins. On the contrary, no wavelength shift was obtained when the immobilized anti-DENV II E protein was introduced to Avidin (as negative control), which was expected considering the fact that the anti-DENV II E protein antibodies have no affinity towards Avidin. Thus, no new layer was formed, and the spectrum remained constant. This experiment shows that the PAMAM-integrated bio-functionalized TOF is selective to the targeted antigenic determinant, DENV II E proteins.

**Figure 4:**
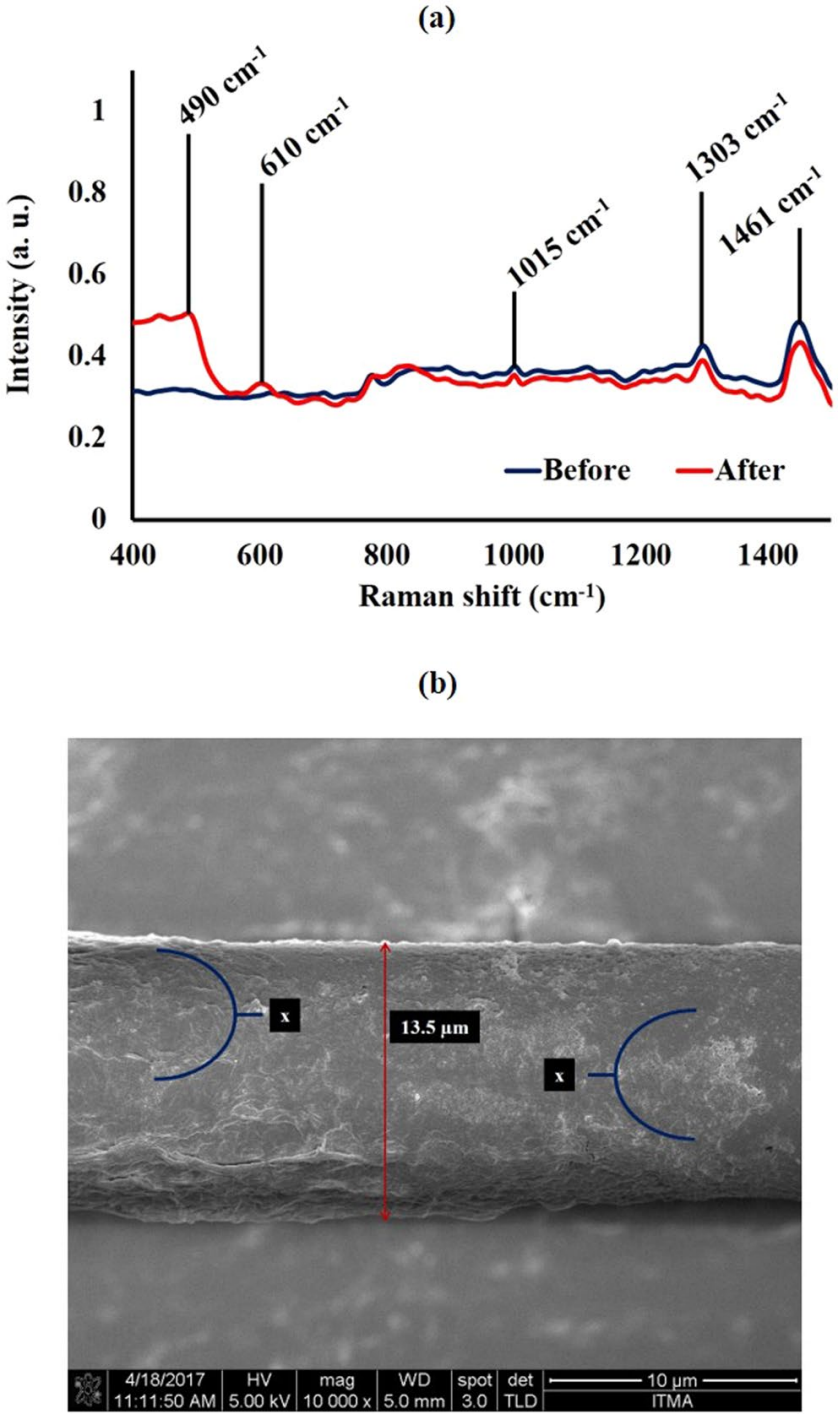
(**a**) Raman spectra taken before and after the introduction of DENV II E proteins to PAMAM integrated bio-functionalized TOF and (**b**) FESEM image taken of PAMAM integrated bio-functionalized TOF after the introduction of DENV II E proteins with 5 kV under 10 K magnification. Thickness of the tapered region here is measured at 13.5 µm.

To characterize the surface of the PAMAM-integrated bio-functionalized TOF after DENV II E proteins were introduced, Raman spectroscopy analysis was performed. In Fig. 4(a), the presence of new band peaks at 490 cm^−1^ and 610 cm^−1^ were observed when compared to the spectrum taken before the introduction of DENV II E proteins. These peaks represent S-S, C-H and C-C vibrational modes. However, peaks at 1015 cm^−1^, 1303 cm^−1^ and 1444 cm^−1^ faced an intensity degradation due to the decrement of free amino functional groups and N-H bonds as a result to the immunological interaction between the antibody and the antigen.

FESEM image of the tapered surface post-introduction of DENV II E proteins was also taken and shown in Fig. 4(b). It can be observed that the entire surface has been covered with a rough and uneven exterior. Another observation to note was the loss of brittle structures which was apparent before introducing dengue E proteins. They seem to have smoothen out as exhibited in areas marked ‘X’ due to the addition of DENV II E proteins. These observations are in line with previous reported studies^27^.

### Sensitivity of the PAMAM integrated bio-functionalized TOF sensor in detecting DENV II E proteins

**Figure 5:**
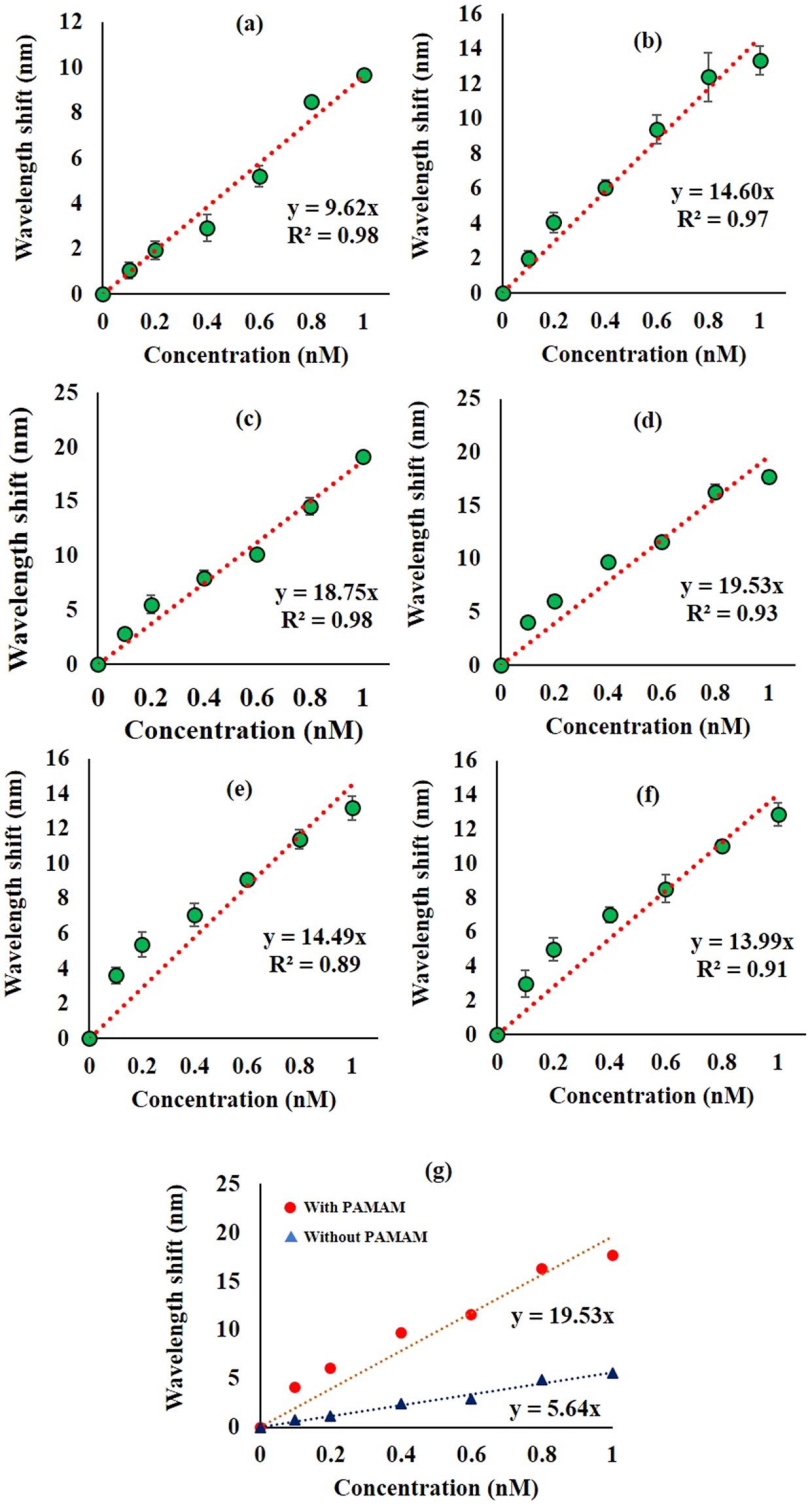
Wavelength shift corresponding to DENV II E proteins at 0.1 nM–1 nM for taper profiles (**a**) P_1_, (**b**) P_2_, (**c**) P_3_, (**d**) P_4_, (**e**) P_5_ and (**f**) P_6_; and (**g**) comparison of sensing performance between P_4_ and control TOF.

All tested PAMAM integrated bio-functionalized TOF showed fair linearity when wavelength shift was plotted against concentration with an R^2^ value of more than 0.88 (Fig. 5). The taper profile which yielded the best sensitivity was P_4_ with a sensitivity value of 19.53 nm/nM. It can be noted here that the overall trend of sensitivity shares a linear relationship with the immersion time until it reaches an optimum point and digresses. The increment can be observed for P_1_–P_4_ with sensitivity of 9.62 nm/nM, 14.60 nm/nM, 18.75 nm/nM, and 19.53 nm/nM, respectively. The following profiles (P_5_ and P_6_) with 50- and 60-minutes immersion time obtained lower sensitivity of 14.49 nm/nM and 13.99 nm/nM, respectively. The data trend of the sensitivity values is also in good agreement with the recorded wavelength shift of each PAMAM TOF when anti-DENV II E protein was introduced Fig. 3(b). This suggests that the sensitivity of the sensor is highly dependent on the antibody load on the sensor. When the sensing performance of P_4_ was compared with control Fig. 5(g), it was observed that the presence of PAMAM increased the sensitivity significantly as compared to the sensitivity value of control at 5.64 nm/nM. The sensing performance and characterization of all PAMAM TOF tested are summarized in Table 1.

**Table 1:** Sensing performance of tested taper profiles.

Taper profile	Immersion time (mins)	Thickness (nm)	RMS	Sensitivity (nm/nM)	Average standard deviation (±)
P_1_	10	8.65	0.86	9.62	0.34
P_2_	20	12.36	1.05	14.60	0.75
P_3_	30	16.40	1.94	18.75	0.56
P_4_	40	21.10	1.95	19.53	0.51
P_5_	50	23.59	2.40	14.49	0.57
P_6_	60	48.42	4.69	13.99	0.51

**Figure 6:**
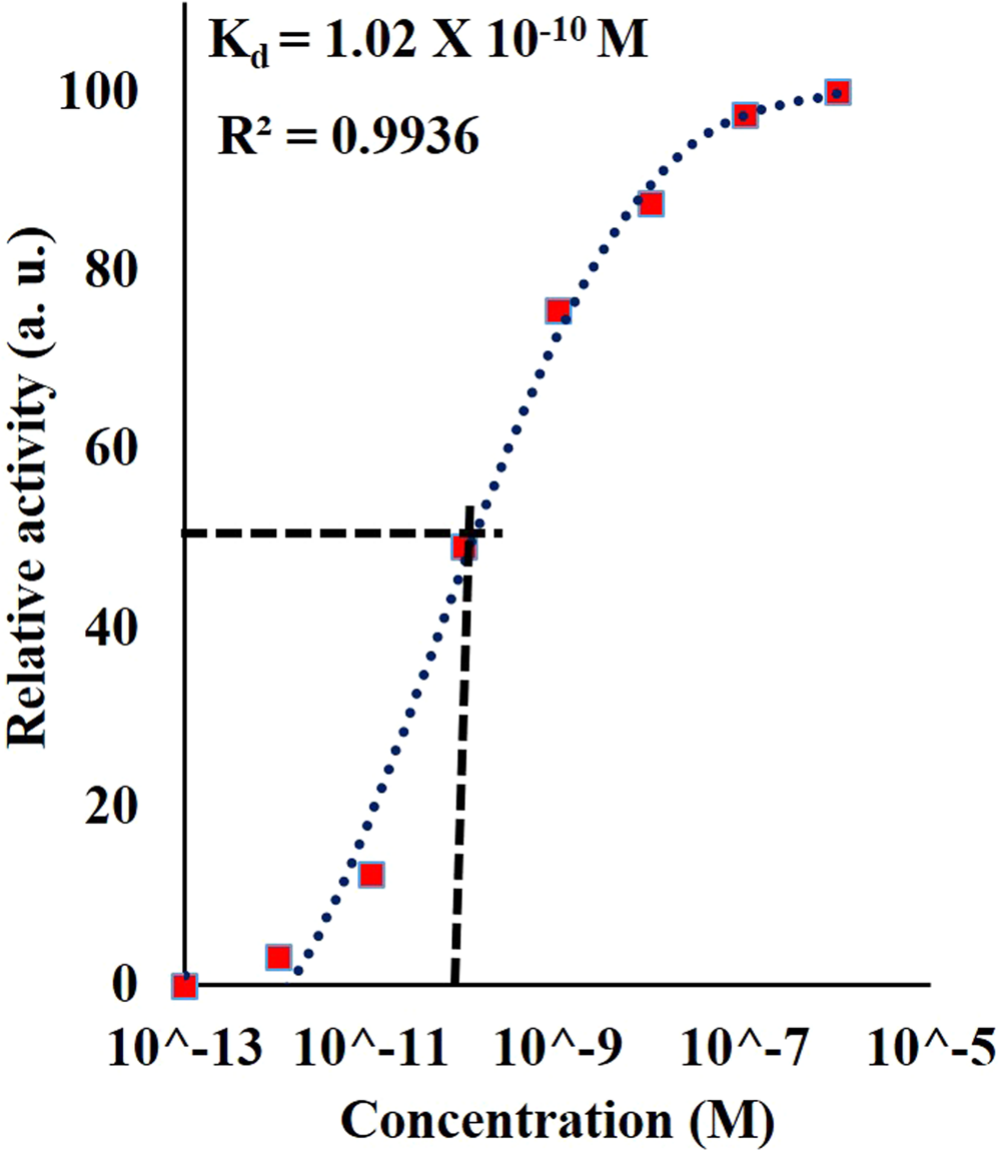
Langmuir isotherm adsorption equation fitting of wavelength shift for PAMAM integrated TOF P_4_ corresponding to concentrations of DENV II E proteins within the range between 0.1 pM to 1 µM. Relative activity here is defined as the ratio of observed shift to maximum shift achieved (Maximum activity = 1).

Limit of detection (LOD) was determined by testing P_4_ with DENV II E protein at concentration ranging from 0.1 pM to 1.0 uM in a triplicate manner. When experimental data was fitted to the Langmuir adsorption isotherm equation as depicted in Fig. 6, LOD was derived at 1 pM with standard deviation value of ±0.02. The affinity of the sensor towards DENV II E proteins was represented by the dissociation constant value at K_d_ = 1.02 × 10^−10^ M; R^2^ = 0.99. The value obtained was lower than what was reported in^25^ supporting positive enhancement through the integration of PAMAM. One of the obvious factors to this would be the larger surface area and more binding sites on the PAMAM molecules. Aside from that, due to the amino functional groups present on the PAMAM dendrimer molecules, the immobilized antibodies were oriented in a tail-on position which was more efficient for antigen binding compared to side-on position which were portrayed in the aforementioned reported study.

## Methodology

### Fabrication of TOF

**Figure 7:**
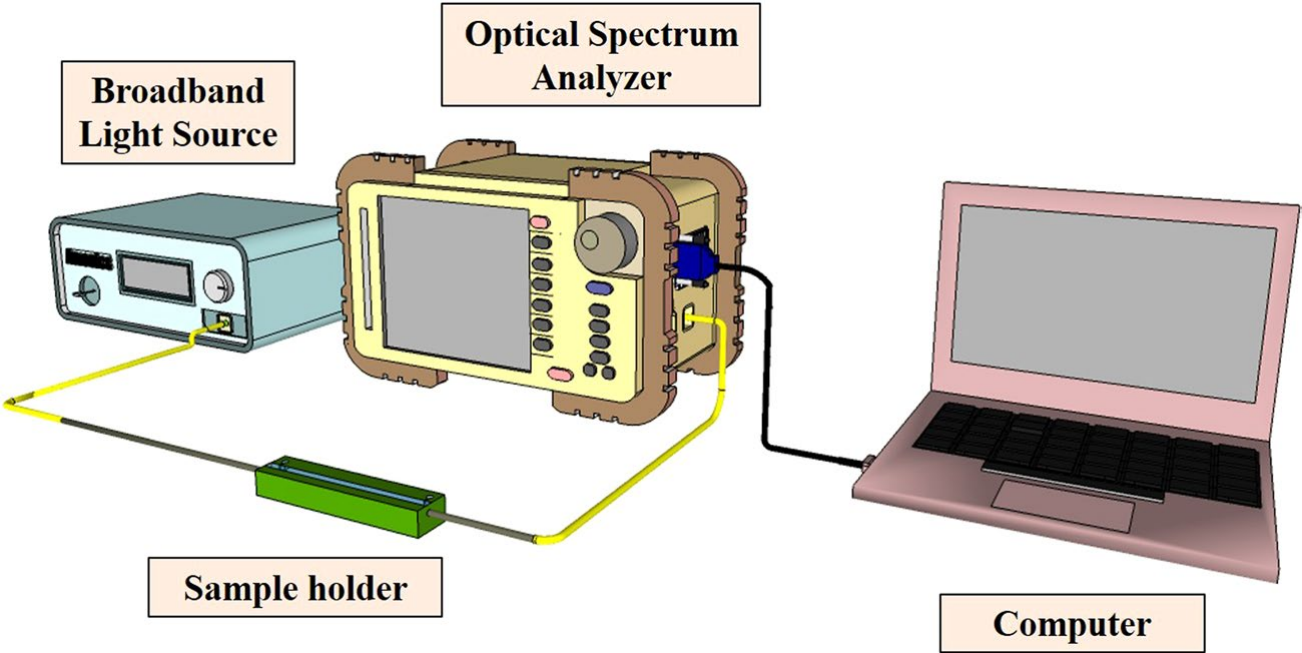
Experimental setup.

Standard single-mode optical fibers (SMF) with core and cladding dimensions of ~ 8 µm and 125 µm were used to fabricate all TOFs used in this work. The tapering of SMF was conducted using the Vytran GPX3000 optical glass processing workstation which operation abides the heat and pull principle. The workstation was well equipped with a real-time system giving users full control over the dimension and uniformity of the tapers. In this work, the waist length and core diameter of the tapered region remained constant at 15 mm and 12 µm following the optimised dimensions in^8^. This was ensured by keeping the heat-power and pulling speed values at 42 W and 1 mm/s, respectively. Following the fabrication process, TOF was secured onto a sample holder with the tapered region hovering in the middle of the sample-well and SMF pigtails were spliced to both ends of the TOF with one end connected to an optical spectrum analyser (Yokogawa AQ 6331) while the other end to a broadband light source (Amonics: 1520 m–1620 m), as shown in Fig. 7.

### Surface functionalization of TOF

To prepare the tapered region for PAMAM deposition, the tapered region underwent a series of functionalization steps. The process was initiated with hydroxylation by incubating TOF in 0.1 M of Sodium hydroxide (NaOH) for 50 mins. This created a layer of hydroxyl groups on the surface of the fiber. After incubation, the solution was drained and the TOF was rinsed 3 times with deionized water. Next, (3-Aminopropyl) triethoxysilane (APTES) was introduced by immersing the TOF in 2% v/v APTES/deionized water solution for 45 minutes. Silane agents, like APTES, act as bridges connecting inorganic materials like the silica surface of TOF to organic compounds. After 45 minutes, TOF was rinsed with deionized water for 3 times and left to dry at room temperature (24 °C) after silanization was completed. To activate the surface, 2.5% of glutaraldehyde (Glu)/phosphate buffer solution (PBS; pH 7) was introduced in the same manner as how NaOH and APTES were. Immersion time was fixed at 45 minutes followed by the rinsing and drying steps at room temperature.

### Deposition of PAMAM dendrimer

Once the TOF was successfully functionalized with APTES and activated with Glu, PAMAM dendrimer functionalization was conducted. Firstly, PAMAM/PBS at concentration 0.1 mg/ml was prepared by diluting PAMAM dendrimer (Sigma Aldrich) in PBS at pH 7, following procedures reported in^28^. The PAMAM/PBS solution was then introduced to the tapered region of the functionalized TOF. Due to the fact that thickness of the active layer plays a part in determining the performance of the sensor, here, optimum immersion time and thickness of the PAMAM dendrimer were determined by fabricating different sets of PAMAM-integrated functionalized TOF with different PAMAM incubation time between 10 minutes to 1 hour. Upon fulfilling the allocated immersion time, TOF was rinsed with deionized water and dried at room temperature of 24 °C. Taper profiles with designated immersion time were analyzed using Raman spectroscopy and AFM.

### Immobilization of antibodies

Specificity of the sensor was ensured by immobilizing anti-DENV II E protein antibodies onto the TOF surface. First, the antibody solution was prepared by diluting anti-DENV II E protein powder (Meridian Life Sciences) in PBS to achieve a concentration of 0.1 µM. The solution was inserted into the sample holder to allow full immersion of PAMAM-integrated TOF for 30 mins. Once completed, the solution was drained out, rinsed and left to dry. To confirm the presence and morphology of antibodies on the tapered fiber, Raman spectroscopy analysis was conducted. Also, output spectrum from the broadband light source was recorded via OSA after each surface functionalization, PAMAM deposition and antibody immobilization for monitoring purposes.

### Detection of DENV II E proteins

DENV II E proteins within the concentration range of 0.1 pM to 1 µM were prepared by mixing DENV II E protein (Meridian Life Sciences) with PBS at pH 7.4. Detection was done by immersing TOF in DENV II E protein solution. The TOF remained incubated in the solution for 15 minutes followed by rinsing and drying of the TOF. The output spectrum for each concentration was taken via OSA. Sensitivity of the sensor was assessed by measuring the resolution and LOD. For sensitivity assessment, each taper profile was tested with different concentrations of DENV II E protein ranging from 0.2 nM to 1.0 nM. Each concentration was tested on a newly fabricated TOF as the sensor was intentionally designed to cater for single use due to hygienic purposes. LOD was determined by taking the taper profile with the smallest sensitivity and testing it with different concentrations of DENV II E protein within the range of 0.1 pM to 1.0 uM in a triplicate manner. To analyse the selectivity of the sensor, another PAMAM-integrated TOF with profile P_4_ was fabricated to test a negative control, Avidin (0.1 µM). All chemicals used in this work were stored in a −20 °C freezer when it was not used while experiments were conducted at 24 °C room temperature.

## Conclusion

In this work, PAMAM dendrimer has been systematically characterized as an enhancement layer to the bio-functionalized TOF sensor for the detection of DENV II E proteins. The best profile with optimum results was P_4_ which obtained a resolution of 19.53 nm/nM and LOD of 1 pM. When compared with past studies, this technique was able to achieve greater sensor affinity towards the targeted determinant with K_d_ = 1.02 × 10^−10^ M. This is owing to the chemical structure of the PAMAM dendrimers that ensures proper orientation of anti-DENV II E protein on the tapered fiber which would increase the absorption of DENV II E proteins. We believe the reported work delivered a promising potential for an improved dengue diagnostic technique.
